# Centering the Margins: The Precarity of Bangladeshi Low-Income Migrant Workers During the Time of COVID-19

**DOI:** 10.1177/00027642211000397

**Published:** 2021-09

**Authors:** Raihan Jamil, Uttaran Dutta

**Affiliations:** 1 University of Liberal Arts Bangladesh, Dhaka, Bangladesh; 2 Arizona State University, Tempe, AZ, USA

**Keywords:** migrant workers, Bangladesh, health, COVID-19, Coronavirus, Asia

## Abstract

A global outbreak of coronavirus (COVID-19) has profoundly escalated social, political, economic, and cultural disparities, particularly among the marginalized migrants of the global South, who historically remained key sufferers from such disparities. Approximately 8 million, such workers from Bangladesh, migrated from their homelands to work in neighboring countries, specifically in Southeast Asia and in the Middle East, and also contribute significantly to their country’s economy. As many of the migrant workers work on temporary visas, scholars have expressed concerns about their physical and psychological health such as joblessness, mortality, abuses, daunting stress, and inhabitable living environment. Embracing the theoretical frameworks of critical–cultural communication, this article explores two research questions: (1) What are the emerging narratives of experiencing realities and disparities among the Bangladeshi migrants at the margins? (2) How the migrants negotiated and worked on overcoming the adversities? In doing so, we have closely examined 85 Facebook Pages (number of subscribers: 10,000-1 million), dedicated to issues of Bangladeshi migrant workers to qualitatively analyze emerging mediated discourses (textual, visual, and audiovisual). Our analysis reveals several aspects, including, (1) impact of job insecurities on migrants and their families, (2) living conditions of and abuses on migrants works, (3) negotiations of mental stress by the marginalized migrants, and (4) how community support helps the migrants to survive during the pandemic.

In 2020, human civilization witnessed an unprecedented crisis; a coronavirus (COVID-19) outbreak affected the lives and well-being of people across the globe in an alarming way. One of the severely affected populations was the migrant workers of the global South, especially those who belonged to lower socioeconomic strata (M. J. Dutta & Kaur-Gill, 2018). Historically, migrant workers in the social, economic, and cultural peripheries (margins) of a society remained sufferers for persistent structural inequalities, discriminations, and strategic negligence from the power structure (M. J. Dutta, 2011). Scholars have noted that the pandemic exacerbated their disparities, particularly in terms of health and well-being, which ultimately resulted in inordinate precarity for the marginalized migrant workers (Mia & Griffiths, 2020).

Globally, mobility of migrant workers nearly doubled over the past four decades (Adhikary et al., 2011). Labor migration is a prominent phenomenon in South Asia as well; approximately one third of the global migrants are from South Asian countries (Weeraratne, 2020). Owing to limited income options and opportunities at home, semiskilled and unskilled migrant workers from various South Asian countries considered working abroad as a potential livelihood strategy (Simkhada et al., 2017). In terms of number of people, Bangladeshi migrants are sixth largest in the globe (Weeraratne, 2020); approximately 8 million workers, migrated from their homeland to work in neighboring countries, specifically in the Southeast Asia (e.g., Singapore and Malaysia) and in the Middle East (e.g., in UAE, Saudi Arabia, Bahrain, Kuwait, Qatar; Bangladesh Bureau of Manpower, Employment, and Training, 2020). In the Middle East, the presence of South Asian migrants significantly affected the host countries, both in terms of demographics and economics. For example, nearly 70%, 50%, and 80% of the total population of Qatar, Bahrain, and UAE are migrants, respectively (Kristiansen & Sheikh, 2014). Similarly, in some Southeast Asian countries such as in Malaysia, one fourth of the migrants are Bangladeshi people (Aryal et al., 2019). In various Asian countries, specifically in the Middle Eastern countries, there is a massive demand for migrant workers; therefore, a large number of foreign workers migrate every year to chase their dreams to improve their domestic economy and quality of life (Weeraratne, 2020).

These migrant workers are making important contributions to both domestic as well as foreign economies. Therefore, during the pandemic, when the migrant workers lost their jobs, the reduced wage income negatively affected their households as well as the macro economy (both in destination countries and at home; Takenaka et al., 2020). As many of the migrant workers work on temporary visas, scholars have expressed concerns about their physical and psychological health such as joblessness, mortality, abuses, daunting stress, and inhabitable living environment. Grounded in the critical–cultural communication framework, this article engages with the Bangladeshi migrant workers’ articulations of disparities, and their negotiations with the adversities, as shared by them in digital spaces (specifically in Facebook).

## Literature Review

### The Coronavirus

In 2020, the world has come to know of a virus called Coronavirus, or COVID-19.^1^ As people already have a general idea of this threat, the researchers have agreed only to share briefly about this virus as part of the contextual framework of the research. A city of China, named Wuhan, saw a cluster of pneumonia cases in December 2019 that were determined to be from an unknown virus strand. Coronavirus is not new to the world and one variant of the virus has been previously seen in 2003 in China that caused the severe acute respiratory syndrome (SARS), and another variant was seen in 2012 in Saudi Arabia as Middle East respiratory syndrome (MERS). The current 2019 strain of coronavirus has not been seen before, and is officially known as SARS-CoV-2 (World Health Organization [WHO], 2020). By August 2020, WHO has reported that 216 countries and territories have been affected by COVID-19 globally, with approximately 26 million confirmed cases and 870,000 confirmed deaths. COVID-19 is believed to have spread from seafood and live animals, but the origin of the virus is still unknown. WHO believes that the current coronavirus is transmitted from the droplets (sneezes, coughs, etc.) of an infected person, or from direct contact with something that has been contaminated with the virus. As such, people who work with live animals, or people who are in close contact with infected individuals (family members, health care workers, people sharing living spaces, etc.) are more at risk than others (WHO, 2020). Accordingly, scientists have strongly recommended maintaining a physical distance (more commonly used as “social distancing”) with everyone till a cure/vaccine has been developed. A few vaccines for COVID-19 has come out in late November 2020 (WHO, 2020).

### Realities of Migrant Workers: A Critical Cultural Perspective

During the COVID-19 pandemic, as previously mentioned, millions of migrant workers across the globe experienced disparities, inequalities, and hopelessness (Mia & Griffiths, 2020). Migrant workers to the Middle-Eastern and the Southeast Asian countries were not exceptions. Critical cultural frameworks, by paying attention to the power dynamics (including governmental, institutional, legal, political, mediated, and economic power structures), contemporary issues (as well as historical) and ideologies, theorize culture as “sites of struggle” (Martin & Nakayama, 2018). Moreover, critical cultural scholars investigate structural dominance/oppression and resistance, communicative phenomena (both mediated and nonmediated), knowledge production processes, human agencies, and organizing potentials toward envisioning an equitable and just world (Ono, 2009). The scholarship calls for reflexive engagements, so that the articulations and narratives, everyday practices at the margins can create communicative entry points for legitimizing situated realities of the underserved in the discursive spaces (M. J. Dutta, 2011). Critical scholars have noted that migrant workers have historically experienced discriminations, prejudices, and unwelcoming gestures, which are intimately tied to their class, gender, racial, ethnic, and religious identities (Martin & Nakayama, 2018).

In terms of health in-access and associated risks, migrant workers are one of the most vulnerable populations; scholars have described their work as 3-D jobs: dirty, dangerous, and demanding (Sweileh, 2018). Scholars further commended to add a fourth dimension, that is, degrading or demeaning, as the voices and agencies of the workers are invisible and/or strategically erased from discursive spaces (Moyce, & Schenker, 2018). Borrowing from critical cultural frameworks in health and intercultural communication such as Dutta’s culture-centered approach and Orbe’s cocultural communication theory, this article examines the marginalized workers’ lived experiences from structural, cultural, and contextual perspectives. Here, cocultural communication signifies communicative behaviors of individuals/communities with little or no societal power. Structures refer to the material- and lived-realities that privilege and/or constrain human communication. Culture is a dynamic, fragmented, and ever-emerging meaning-making process that create avenues for the constructions of health and well-being. Agency connotes the capabilities of peoples to negotiate with structures, and their ability to discursively challenge their ability to be healthy. The term context refers to local environments and surroundings where people enact their voices and agencies. Finally, researchers’ engagements in reflexively listening to the voices of underserved populations, according to critical–cultural theorists, potentially create avenues for social transformations (M. J. Dutta, 2008; Ford & Yep, 2003; Orbe, 1998). Although precarity of migrant workers, especially those who are from the Global South, is addressed in extant critical–cultural scholarship; their lived experiences and agentic negotiations during an unprecedented pandemic necessitates fresh scholarly attentions. From methodological perspectives, we examined the existing digital discourses that are created or shared by the migrant workers themselves (i.e., we did not provide any prompt for discussion or participation to the workers). Scholars noted that a “democratized” access to digital spaces often aids cocultural populations to enunciate counterhegemonic discourses, which, in turn, potentially yield a candid and thick data as well as enrich the overall research processes (Kavanaugh & Maratea, 2020).

### Structural, Cultural, and Contextual Challenges

The critical lens helps us understand the unequal distribution of and access to basic resources/necessities, structural disparities, and violence (including material and communicative) as experienced by the migrants (individually as well as collectively; Sargent & Larchanché, 2011). In migrants’ health context, a critical health communication perspective investigates various structural disparities and cultural challenges, such as inadequate living condition, unhealthy work environment, psychological stress, as well as limited knowledge of local language, and access to health information and/or services (Jamil & Kumar, 2020; Sargent & Larchanché, 2011). Furthermore, migrant studies scholars noted that, lack of compassionate and humane health policies, inadequate legal provisions and welfare supports, discriminations and violence (such as microaggressions and hate), and uncertain financial scenario pose challenges to the migrants, who being one of “the most vulnerable subpopulation” socioeconomically, often are deprived of adequate social support during the pandemic (Alahmad et al., 2020). Some of the key challenges are discussed below.

#### Legal Barriers

Owing to inadequate legal protections and weak legal documentation (by job agencies/companies), many migrant workers often experience exploitations and unfavorable legal consequences (Takenaka et al., 2020), including in-access to necessary basic resources and services that diminished their chances of securing meaningful health care access (Mia & Griffiths, 2020). Moreover, legal provisions in destination nations such as *kafala* system, many migrant workers in the Middle Eastern countries historically experienced difficulties in assimilating with the host cultures (Jamil & Kumar, 2020; Karim et al., 2020). In other words, weak legal position, nature of the job (often short-term/ temporary/contractual), less welcoming environment abroad, have all contributed to increased health (and well-being) disparities for marginalized migrant workers (Adhikary et al., 2011).

#### Language and Information Barriers

Language barriers, such as limited abilities to speak, write, and understand local/host languages, often negatively affected the quality of health care services the migrant workers received in the destination countries (Sweileh, 2018). Again, information-poverty, specifically, poor knowledge about the health care system, health coverage, and policies of the destination countries, prevented semiliterate and less-educated migrant workers to seek health care and meaningfully access health services as well as claim benefits; for instance, oftentimes they presumed health care abroad as unaffordable, and therefore, often preferred to wait, or go back to their home countries for treatment (Ang et al., 2017). Thus, low educational levels and information asymmetry along with inadequate language skills, negatively influenced the overall quality of lives of the migrant workers.

#### Financial Challenges

Severe financial and economic difficulties along with social exclusion/rejection in both home and destination countries affected the lives and health of many vulnerable migrant workers (Mia & Griffiths, 2020). Previous research showed that family members of migrants often solely or largely financially dependent on immigrants’ incomes (Mia & Griffiths, 2020). On the other hand, while trying to save their jobs, migrant workers sometime had to consume their reserve funds to ensure survival of their family members at home (Karim et al., 2020). Moreover, scholars noticed that during the pandemic, social acceptance of marginalized migrant workers fell drastically; in some cases, home countries, even closed their borders to migrant workers, their own citizens (Mia & Griffiths, 2020).

#### High-Risk Living Environment

Experiences adverse occupational and living environment, and participation in risk-taking practices (e.g., taking risks for earning money in a competitive work environment), often make migrant workers are a high-risk population in terms of health and safety (Simkhada et al., 2017). During the pandemic, many migrant workers quarantined in unhygienic and cramped living spaces. Some news reports suggested that up to a dozen migrant workers were placed in a single room of dilapidated houses, where the availability of essential resources such as hand sanitizers, soaps, and sufficient water were scarce (Gasana & Shehab, 2020; Kristiansen & Sheikh, 2014). Moreover, impossibilities in implementing social distancing and other measures (WHO recommended) and social habits of the migrants (influenced by collectivistic cultures) potentially made their living spaces epicenters of COVID-19 outbreak (Karim et al., 2020).

#### Mental Health Aspects

Moreover, scholars noticed that the mental health of many migrant workers deteriorated owing to their concerns about their uncertain income, health of family members at home, and fear of deportation (Gasana & Shehab, 2020); moreover, many migrant workers lost their friends and family members. (Simkhada et al., 2018). As they could not travel internationally, from cultural and religious perspectives, their inabilities to participate in funerals/religious rights negatively affected the mental health of many migrant workers and their families (Mia & Griffiths, 2020).

Embracing the theoretical frameworks of critical–cultural communication, this article explores two research questions:

**Research Question 1:** How did Facebook contents brought forth Bangladeshi migrant workers’ narratives of disparities and lived-realities?
**Research Question 2:** How the underserved migrants negotiated and worked on overcoming the situated adversities?

## Method

Centering around the low SES migrant workers’ experiences with the COVID-19 global pandemic, we wanted to study their lived realities during this unprecedented time. Our goal was to find out the emerging narratives of negotiating disparities and agentic potentials among the Bangladeshi migrants at the margins. This pandemic shaped our research agenda as well as influenced our research methodology. The pandemic scenario prevented the authors to go to different locations and observe interactions in-person, or conduct face to face in-depth interviews to collect data. As a result, digital ethnography and socially mediated resources became invaluable to us. Digital ethnography aided us to study the contents (e.g., texts, videos, and images) about migrant cultures, behaviors and communications in the digital spaces. Such an approach of conducting research opened up avenues to accessible way of collecting data during the pandemic (Góralska, 2020). The data we have collected using electronic means are detailed, and also supports the call to acknowledge the richness of electronic data in a rapidly growing digitized world (Lupton, 2019; Lupton & Watson, 2020; Ramsetty & Adams, 2020).

Our research participants were low-income migrant workers who live and work in two geographical regions of the world—Southeast Asia and the Middle East. These are two regions that have historically witnessed a high number of South Asian migrant workers; a large number of them were Bangladeshi migrant workers. The linguistic proficiencies and past research experiences of the authors made it a pragmatic choice to focus on community members who spoke Bangla/Bengali. The researchers are fluent in the Bengali language, and have previously worked with communities that included both migrant workers and Bengali speakers. So, the experiences were useful in working with this context as well, as the Facebook postings were mostly in Bengali, and others were in English. We investigated online Facebook communities of Bangladeshi (groups and pages—“groups” in future references) migrant workers who live and work in those two regions. Facebook had been a preferred choice for migrant workers to share and voice their experiences and concerns over the years because of its ease of use and accessibility (see Dehkhoda et al., 2020; M. J. Dutta, 2018; Kumar & Jamil, 2020). We also searched for similar publicly available sources of information on other popular social media sites such as Twitter, Instagram, and Snapchat. But no other platforms had as much detailed and voluntary communication and self-reporting engagements from marginalized migrant workers as Facebook.

The authors searched for relevant pages on Facebook, based on Facebook’s own search, Google search, and the authors’ existing social/research connections. These Facebook groups/pages act as communicative spaces for migrant workers to share their thoughts, concerns, feelings, and so on, and can be a rich source of getting an insight into their everyday lives. People have used these groups for voicing their concerns, and also to find others in similar conditions to talk to. We then made a list of all possible sources of relevant Facebook pages, and then compiled them into a primary list. As the list grew very long, we decided to narrow our list down by only choosing groups that had over 10,000 followers/members in them. For this research, we have observed 97 Facebook pages that focus primarily on Bangladeshi migrant workers’ experiences. The lowest number of followers/subscribers on this list was 10,049 and the highest number was about 1.4 million. We studied the groups’ postings in a chronological order, and went as far back as the beginning of March 2020 (and sometimes the end of February)—a time when COVID-19 became more prevalent worldwide. While engaging with the socially mediated contents, we wanted to observe and understand how the community members were negotiating with the issue of COVID-19 in their lives (individually as well as collectively). We saved those posts using “collections”—a folder like feature built inside Facebook—that were linked with COVID-19 and related matters. Both the researchers compared their “collections” to check for intercoder reliability. Once we felt comfortable with our categorizations and rationale behind them, we went ahead and analyzed our collected data till we reached data saturation (we had 41 categories of posts). Inside these categories, we had over 550 items saved. These COVID-19-related items included direct posts, comments, responses, shared materials, audio, video, and other external links.

As researchers, we wondered about one thing in particular about the Facebook posts. With so much stress and struggles of everyday living, how did the users manage to have internet access? Without any direct interactions with the groups’ members, we could not directly ask that question. But based on our previous experiences in working with members of similar communities, personal experiences, and relevant literature (Kaur-Gill & Dutta, 2017; Kumar & Jamil, 2020), we know that the price of Internet has become cheap globally, and that migrant workers consider this their primary mode of communication (Voice Over Internet Protocol—VoIP—audio/video calls) with their family members. As such, many consider Internet usage as a necessity. In some cases, landlords or employers provide a wireless internet access for a whole building and anyone living in that building case are allowed to use the service. In other cases, low-income individuals use something called Facebook Zero, which is a text-based service that Facebook provides free of charge in collaboration with mobile service providers in different countries. So, internet use, and more specifically Facebook usage is common among migrant workers in many different countries. We went through the details of each relevant post, its comments, replies, and also went through each shared content posted by the groups’ members. For example, when someone shared a video clip, we made sure to watch the whole clip, and read the associated comments and responses. As we read, watched, and listened through the relevant content, we took notes, and also wrote field notes and memos, that later aided us in analyzing our data and make sense of ideas.

The data thus collected from observing these socially mediated communications in those open-for-all public platforms were analyzed using constructivist grounded theory (CGT; Charmaz, 2006). In a CGT, researchers “study how—and sometimes why—participants construct meanings and actions in specific situations” (Charmaz, 2006, p. 130). This aligned well with our research objective, as the CGT keeps the lived experiences of the participants very much into focus. The first stage of CGT focuses on creating as many classifications as possible—known as open coding. To some extent, our initial Facebook “collections” served that purpose, although we created more when going over them back and forth. For example, when migrant workers talked about how the financial uncertainty is causing them stress, we open coded that as “mental stress,” and when they talked about taking their own lives as the stress was becoming unbearable, we open coded that as “suicidal tendencies.” Open coding was followed by an axial coding process, where the codes were derived based on their interconnectedness (conceptual clusters). For example, the open codes aforementioned were categorized into “Mental health: Negative aspects” during an axial coding stage, where all the related codes on mental health impacts were connected. In the third and final stage of the data analysis, selective coding, these conceptual clusters were analyzed and woven into a “meaningful theoretical framework, so that the cultural and contextual meanings of the participants’ narratives emerge organically” (Kumar & Jamil, 2020, p. 1727).

## Results

### Job Insecurities

One of the most unfortunate and large groups of people who lost their jobs due to the global coronavirus pandemic were the low-income migrant workers from all around the globe. This research focused on low-income migrant workers from Bangladesh across the Middle East and Southeast Asia, in low paying jobs (such as construction sites, janitorial, oil fields, etc.). With a virtual lockdown and/or shutdown of many of these industries, millions of workers lost their jobs/earnings (Sumon, 2020). One report stated that “foreign and domestic agencies were anticipating more dire times ahead, and the Bangladesh government should increase coronavirus relief support” (Chowdhury, 2020b). Additionally, a severe decline in global tourism and the aviation industry also led to a chain of events that led to huge job losses particularly among the blue-collar workers. By June 2020, over 1.4 million Bangladeshi workers had lost their jobs, and over one hundred thousand workers were forced to return to Bangladesh (Chowdhury, 2020b). Additionally, the several hundred-thousand workers who were visiting Bangladesh could not return to work in their respective workplaces abroad (Islam, 2020).

The low-income, especially the irregular-income (seasonal/temporary jobs) earning migrant worker communities across the globe has suffered more harshly due to the pandemic than many other communities. These are people who earn a very small salary (or wages) depending on their work done. And in many cases, even then, the companies that employ them sometimes pay their salaries keeping a backlog—meaning that very rarely all salaries are cleared off on time. It is common to find workers who have not received their salaries for several months (M. J. Dutta & Kaur-Gill, 2018; Kumar & Jamil, 2020). So, for many workers, even with a job, their situation is similar to others without jobs.

It is not only the actual job loss for many of our research community members, but also the possibility of losing one’s job acted as a constant fear. “Workers in Malaysia,” for example, “are scared of the prospect of losing their jobs more than the coronavirus infection itself and a majority of the people are spending their days worrying about their possible unemployment” (Ahmed, 2020b). Additionally, workers who are losing their jobs are also worried about their being able to support their families back in Bangladesh. In many instances these workers are the sole breadwinners in their families. So, a loss of their jobs/earnings directly affects the lives of many family members. The story of Bidhan Chandra Sharma, for example, got a lot of traction on Facebook groups, from a report published on the BBC News Bangla website (“Foreign workers’ unemployment,” 2020). Sharma, a Bangladeshi migrant worker, worked at a hair salon in Saudi Arabia. One of the first businesses to shut down after the Saudi government declared a lockdown due to the pandemic were the hair salons. Many like Sharma not only lost their jobs but also could not afford to eat 3 times a day after a few months of being jobless—leaving aside sending money home to their families. Also, people did not know when or if they would be able to get their jobs back. Their employers do not contact them or give them any money. Sharma was the only income generating member of his family of eight living in Bangladesh, who are now borrowing money from others just to survive. One migrant worker related to this news on Facebook and wrote as follows:

I was working outside of Riyadh [Saudi Arabia], and I do not have work for the past five months. The company is not paying our salaries, and also not paying us for our food [some companies pay for food in the Middle East]. I am bringing money from home and eating. We are in deep trouble.

Another person, who is in a similar position, wrote, “The company is giving salary after three-four months. So, I am not working. No work.” This is followed by another comment—“There were five of us before, but now three has moved out [as they had no money]. Please come and visit us if you can [to see our distress].” Another person wrote,

We have been sitting idle for the past six-seven months, without any work. The company sometimes gives us a little bit of money for food, but that runs out after buying water. Many people are bringing money from home [Bangladesh], but now those options are also getting limited. There are people who have been without work for the past 10 months . . . [and can neither stay nor go back].

One report stated how 87% of the migrant workers, who lost their jobs due to the pandemic and are returning home to Bangladesh, do not have any source of income in Bangladesh (Ahmed, 2020a). Of this group, about a third can survive for roughly 3 months on their current savings, and over a half have expressed their needs for financial assistance. Roughly half of these workers have returned from the Middle Eastern countries and the others from the Southeast Asian countries, the Maldives, and Italy.

### Living Conditions and Abuses

We have noted above how several migrant workers were forced to move out of their already congested living arrangements, due to financial struggles caused by COVID-19, and had to stay with friends or relatives. But at the same time, there are many social media postings about how more people are trying to stay together in order to save on rent payments. Not only that, many migrant workers were “forced to stay in local mosques and survive on charities provided by the mosques” (Ahmed, 2020c) and other residents. A typical labor camp where low-income workers live, houses a dozen or so people in one small room with multiple bunk beds, and there are also reports of more people staying together (Cornwell, 2020). Millions of foreign workers are going through difficult living conditions among other stressors. In the UAE and Bahrain, for example, empty buildings (some are under construction) or schools are being used to rehouse migrant workers from overcrowded living conditions (Cornwell, 2020), as crowded places are more prone to coronavirus outbreak. One person struggling through such harsh conditions said,

We do not have our employer beside us, nor our High Commission [of Bangladesh]. Sometimes I wonder if luck has deserted us as well. We are living in a world where no one is standing beside anyone. No one is showing any compassion or support. Life has never been more difficult.

A widely circulated video on Facebook shows how 15 workers were ushered out from a single room, furnished only with multiple bunk beds, for COVID-19 testing in Saudi Arabia. There have been many videos on Facebook—although they were of people from multiple different nationalities and locations, and not only Bangladeshis—that show how low-income workers are struggling for accommodation. One specific video claimed how people from an African country living in the UAE were being forced to live in the streets (and nearby desert) as they have been evicted from their homes, apparently for not being able to pay their rents.

Living conditions might be worse for female migrant workers. Many social media posts, along with mainstream news sources, have reported how female workers’ working conditions have deteriorated due to the COVID-19 situation. Hubbard and Donovan (2020) reported how nine female migrant workers were locked in a small bare room with only a few thin mattresses when they lost their jobs in Saudi Arabia working as housekeepers. Some of them have been locked up there since March 2020, and one of them—who is 6 months pregnant—has not received any form of medical care. These women receive food once a day, and they have no idea when they will be able to get out of this “prison” or go home. Female migrant workers from Bangladesh have similar stories to share. Multiple such workers, who worked as domestic helps in Saudi Arabia, said, “we would rather beg in Bangladesh to survive, rather than go back to Saudi” (Ovi, 2018b). Although this is not from the COVID-19 timeframe, it is important to listen to these voices, as things have only worsened during the pandemic (Azeez et al., 2020). Millions of women from the Indian subcontinent, the Philippines, Indonesia, and several African countries are employed as housekeepers and nannies in the Arab region (Hubbard & Donovan, 2020). With the COVID-19 lockdown, the women have been forced to work harder than before. Many of these women were already working around 16 hours a day without any days off. Now with most house members staying in, and working from home, they are having to work virtually nonstop (some have claimed as many as 21 hours a day) for all the house members, including sterilizing entire houses with chemicals that are sometimes harmful for their own health (Begum, 2020). Many workers have written about getting a reduction in their salaries with their employers using coronavirus as an excuse. Many others’ jobs have been terminated without any payments. In Lebanon, for example, local employers simply dropped off their female workers in front of their respective embassies in Beirut without any payment of any form (Hubbard & Donovan, 2020). “I worked in Jordan for a year and a half under difficult conditions where the houseowner made me sleep on the floor and she would kick me awake. I don’t want to return to Jordan,” said Safa, a female domestic worker who lost her job due to the pandemic, and was forced to return to Bangladesh (Bangladesh United Nations Network, 2020). Female workers of Bangladesh who work abroad, however, do not always feel comfortable talking about their experiences/ terminations from their foreign work due to the fear of social taboos, and their family members’ retaliations. A key reason for that is the nature of the abuses they face while working abroad, which in many cases are sexual in nature. Growing up in parts of Bangladesh and India that share multiple cultural, linguistic, and social similarities, such practices of not sharing information are very common, and have been witnessed several times by the authors. Additionally, there has been multiple reports about such abuses in national newspapers (Ovi, 2018a, 2018b)

Even when the community participants were not evicted, forced to cramp in small places, or left at the embassy doors, they were victims of market price increases on necessities. As more countries struggled to limit the spread of the coronavirus, more businesses were shutting down, and countries were getting into lockdown situations. Apart from the basic necessities and emergency services, countries around the globe came to a virtual standstill. As a direct result of this, many people got into a buying frenzy of basic necessities, such as milk, eggs, bread, oil, toilet paper, among other things. But according to individual posts and news sharing, many dishonest business owners increased the prices of these commodities, citing multiple excuses (higher demand, the extra cost of shipping Bangladeshi products into the stores, etc.; M. M. Uddin, 2020). So, on one hand the migrant workers were not getting paid or did not have much money in hand, and on the other hand the price hikes meant that they could not buy what they needed and as much as they needed. One participant working in Malaysia wrote, “When my company told us there will be a lockdown for the next 14 days, I went to my local Bangladeshi store. But some dishonest businessmen had already increased the prices of vegetables, chicken, and fish” (M. M. Uddin, 2020). He felt the prices were almost doubled overnight. Another participant shared that although other stores in Malaysia did not reflect any price hikes, only Bangladeshi store owners increased the prices of things. Another frustrated Bangladeshi worker said, “[when I went to a Bangladeshi store], I saw there is price increase of four to five Ringgit [US$1 = Malaysian Ringgit 4.17] on all fruits, vegetables, fish, chicken, and meat” (M. M. Uddin, 2020). So, some businessmen were abusing the situation, and the limitations of the migrant communities, for their own advantages.

### Mental Health

The pandemic has hit the migrant worker community very hard across the globe. Whether it is the Southeast Asian countries or the Middle Eastern countries (our research focus), workers have reported severe financial, physical, and mental/psychological distresses. Malaysia, for example, is home to one and a half million Bangladeshi migrant workers who are mostly out of work since mid-March due to the lockdown implemented by the government (Islam, 2020). A majority of these workers have been sitting idle in their residences hoping for the lockdown to be lifted so they could send money home to support their families in Bangladesh. But that has not been possible, and the stress arising from that has been explicit in their comments. “How would I eat, and pay my rent at the end of the month?” asks an individual in a Facebook post, expressing worries about sending money home. Another person wrote,

My mother is home sick, and she cannot go to the doctor because I have not been able to send any money home in the past months. I worry that if something happens to my mother, I would never be able to forgive myself. But I do not have any income to send home.

On May 4, the Malaysian government announced that some workers might return to work only if they could get tested for the coronavirus (Islam, 2020). The workers who did not have a specific insurance—and one million did not—would have to pay out of pocket for it. The cost of a test is RM 360 (US$ 86; Islam, 2020), and many of our community participants earn around RM 1,200 (US$ 288) or less a month (“Migrant workers can’t afford,” 2020). This further compounded the stress the workers were already in. On one hand, people needed the work, the income, for many reasons, but on the other hand, they needed the money (which they did not have) to get the work. This is the grounded reality of the migrant workers’ lives in Malaysia, and many other countries. The structural constraints limit workers from actually getting the job, whereas such barriers are framed in terms of doing more tests for the safety of the people of the country. Moreover, many workers who went for a visit back to Bangladesh expressed their worries about returning to their workplaces due to the lockdown, testing expenses in Bangladesh and the foreign country, and the availability of flights to/from the destinations. The uncertainty of returning to work, compounded with the real fear of not being able to earn a living for the family has been shared across many Facebook groups. “Another sad fact is that several thousand migrant workers who went home are now spending their days in extreme stress, not knowing when they will be able to return to their jobs, or return at all,” says Islam (2020).

Additionally, many workers who have worked before or during the lockdown are worried about not getting paid. There are questions of stress and uncertainty regarding this as well in the Facebook groups. One participant posted, “Brother, now that we are not working, will the company still pay us?” Another participant replied, “Our work is going on as usual. Will our company also shut down?” These fears easily translate into the health and wellness of not only the individuals working (or sitting idle) but also the lives of each family member they are supporting back home with their incomes. One individual shared a Malaysian government’s press release with content written in Malay, that apparently talks about helpline numbers people can call, if they are forced to work during the pandemic, or if they are not getting paid for their work. However, many workers cannot read Malay and do not know the actual content of the message. As one participant wrote, “My company is not paying me brother, what can I do? I do not speak Malay.” The added confusion and lack of clear communication, along with the linguistic barriers, have added to the stress of many migrant workers.

In some extreme cases of mental distress, there has been news of people wanting to commit suicide, and actually committing suicide. Apart from the worries of supporting self and families, the boredom and stress of staying with many others in a closed/small place for a long period of time, also contributed to such feelings of despair. One person shared his feelings—“I am writing this from Mecca, and I cannot take it anymore brother. We are locked in a room for two months and 18 days, and only Allah knows when Mecca will be free from corona.” As noted earlier, many of the living accommodations for the low-income migrant workers abroad range from 10 to 20 people in one room with multiple bunk beds where they sleep and also keep their belongings. Another person wrote, “I really cannot take this anymore. Is there an underground way to travel from Mecca to any other city?” The severe frustration is obvious from this question, as the person is perhaps thinking that underground roads/tunnels are safe for traveling during the pandemic. “People in Mecca are really suffering, what can we do?” wrote yet another person. A community member, perhaps after forsaken all hope, wrote, “We are all in the same situation, and do not even know who we can share our sorrows with. There is no one to talk to. Only God knows.” One specific post shared the news of a migrant worker named Mohammed Ariful Islam, who was working in Malaysia, and was laid off from work during the pandemic. He could not support himself, and also could not send money home. Islam committed suicide by hanging himself from a ceiling fan. People close to Islam think he committed suicide as he could not bear the pressure of his family asking him for money, which he did not have (M. M. Uddin, 2020). So, the combined stress of work, life, and living drove him to take his own life. People like Islam are perhaps not alone. Others have shared videos of how people were protesting the Bangladesh government’s lack of support for them (in front of the embassies abroad), by expressing their anger and frustrations with slogans/chants of suicide. Yet others have written about their grief and stress of not being able to join the final religious/cultural rituals of their loved ones due to the lockdown situations in their home and work countries.

### Community Support

As discussed above, the community participants of our research project have been struggling with multiple aspects of life and living for a long time, which got even more complicated due to the COVID-19 outbreak. But even then, support structures have developed in many countries that are trying to assist the workers as much as possible. Saudi Arabia, for example, hosts over two million Bangladeshi migrant workers, and there are many doctors working in Saudi Arabia (Ahmed, 2020d). A panel of such doctors have come together and have asked the Bangladesh embassy there to start a free medical advice/counselling service for all Bangladeshis living in Saudi Arabia in Bangla language. A medical panel has accordingly been formed, called *Probas Bandhu* Call Center (Migrant Friends Call Center), who is working tirelessly to provide medical advice to anyone in Bangla language (Ahmed, 2020d). As many workers live in remote parts of the country, and as the country has been under lockdown and partial curfew for the past several months, it has been very difficult for many migrant workers to physically go to a medical facility. On top of that, it is not guaranteed that the workers spoke Arabic (the main language of Saudi Arabia), or English, or that the medical facilities had translators or doctors who could speak Bangla. So, the availability of doctors’ medical support over the phone in a native language has been a source of immense support for the workers. Over 65 doctors have registered for this service through the Bangladesh government’s coronavirus official website at www.corona.gov.bd. This service has been very well received by the Bangladeshis (Probas Bandhu Cell Center, 2020).

A similar community support initiative has been taken by another organization called the “Dhaka Medical Center.” This organization started to assist families of severely affected migrant workers with reduced medical testing fees, financial support, and Eid gifts (Chowdhury, 2020a). Eid is the name of the religious festivals that Muslims around the world celebrate. In 2020, both the Eid festivals happened during the COVID-19 pandemic. Dhaka Medical Center has committed to continuing their services till the pandemic is over. In a situation where literally, millions of people are struggling to put food on the table, let aside seek medical assistance, such a service acts as a beacon of hope and example for others to follow (Chowdhury, 2020a).

Not only organizations but also individuals have come forward to assist others in need. A couple in Singapore has become a social media and news outlet favorite when they started to give out *Iftar* during Ramadan (the evening meal), and other dry foods from their own resources (M. S. Uddin, 2020). Mr. Kabir Hossain (a Bangladeshi-Singaporean) and his wife Nooriya (an Indian-Singaporean) happened to see one restaurant in Singapore giving out free food. Mr. Hossain initially donated some money there, but eventually he started to donate food with his wife. They arranged for a small truck and started with their charity work from one end of the city to the other, sometimes from morning till midnight. Watching their dedication, others have also come forward to support this couple (M. S. Uddin, 2020). A similar initiative was also reported about a migrant workers’ group from Bahrain. Here, two brothers from Bangladesh named Alauddin and Kamal took it on themselves to share as much food and basic necessities as they could to the less fortunate workers who have lost their jobs due to the COVID-19 outbreak. So far, they have supported about 300 migrant workers.

Much like the Hossains, the Migrant Workers’ Center in Singapore has also been trying to assist the migrant workers who have been struggling to make ends meet during the pandemic. On their Facebook page, they have written,

Over the weekend, we packed and distributed essential hygiene, personal care and dry-ration food items. . . . More than 7,500 hygiene and personal items like masks, hand sanitizers, bath soap, and more than 15,000 food items were delivered to. We are proud and happy that the migrant brothers we’ve met this weekend continue to keep strong and positive during this period despite being faced with disruptions, inconveniences and uncertainty in some instances.

Apart from these initiatives, there have been other posts on Facebook how community groups in Bahrain, Saudi Arabia, UAE, Singapore, Malaysia were trying to support their communities in any ways they could. During the Eid days, a community in Bahrain gave away “Eid gift packs,” plus some food traditionally eaten in Bangladesh during such festivals. A sweet shop in Saudi Arabia similarly shared sweets with the people in distress, along with some Eid gifts. A video was shared by community members in Malaysia that gave clear instructions about how to get food and support from a not-for-profit organization called *Bhalobashi Bangladesh* (Love Bangladesh) that is delivering food to people in need. This organization would deliver food to anyone in need, free of charge. There were many other examples of how community members got together, raised some funds individually, or from local businesses and local affluent people, and then made food packets for distribution. Many such initiatives made it a point to go to the labor camps, or dormitories, where the workers stayed in groups, and delivered the food there. In the UAE, for example, two separate organizations (and many individuals) collected funds (once a month for 3 months), made food packets, and delivered them to camps and other residences of low-income migrant workers. These packets had enough food to last an individual for a month. Many of these efforts were also supported with news and postings about job vacancies, emergency numbers, and free food services that people might find useful. So, during times of need, just as hardships got worse for many, hope, help, and ways of community bonding also surfaced to counter those hardships. Grassroots structures developed that provided material, psychological, and communicative support to many people in need.

## Discussion

Espousing the critical cultural frameworks, this research seeks to foreground the otherness and different realities of the migrant workers towards opening up avenues for voicing the narratives of in-access (M. J. Dutta, 2011). Otherness here is characterized by attributing a “less” social stature/power characteristics (typically negative) to the migrant worker community members, to situate them outside of the perceived normative group (Brons, 2015). Cultural insiders as well as their articulations and agencies (the pivotal components of the research engagement), are central to create a communicative infrastructure toward ensuring human rights and dignity (U. Dutta, 2015). One of the key contributions of this article is the theorizing of the precarity of migrant workers and their experiential realities of everyday living, by connecting the intertwined interplays of structural, cultural, and contextual complexities in the backdrop of a global pandemic. The emerging narratives of marginalized workers foregrounded the role of power and structures that worked in multiple layers (e.g., at the micro-, meso-, and macro-level) in the realm of agentic negotiating uncertainties and health seeking. Health of underserved migrants is therefore composed of their (individual and communal) vulnerabilities in foreign spaces amid global neoliberal functioning. Interactions between culture and structure were also revealed through the voices of migrant workers, as structural absences (e.g., discrimination, in-access, and exploitations) and communicative absences (e.g., voicelessness and isolation) constituted as well as reinforced each other.

Marginalized migrant workers, being at risk of disparities, negotiated with several adversities, including financial uncertainties, insufficient medical attention and facilities, in-access to health care, along with limited affordability to avail necessary care, and services (Mia & Griffiths, 2020). Moreover, oftentimes, they experienced rejection and negligence from their recruiting companies and agencies, which yielded mistrust and hopelessness among the low-wage workers (Lee et al., 2014). Again, the state of social isolation (if not abandon, in some cases), lack of language-proficiency (and meager access to language interpreters), and their inability to meaningfully communicate with public servants and medical professionals exacerbated their conditions of marginalization (Moyce & Schenker, 2018). In other words, precarious employment, inadequate legal support, material scarcity and poverty, limited access to health care, insurance, and information negatively affected their physical and mental health.

Scholars observed that communicative absences and contextual factors affected the health and aggravated health risks among migrant workers, especially those who belonged to lower socioeconomic strata (Sönmez et al., 2011). During the pandemic, migrant workers and their families were often ignored by the policymakers and health care providers both in the home and destination countries. As many of the migrant workers were less-educated (in terms of formal education), they often experienced lack of access to information about the laws and policies of destination countries, including the health-related ones (Ang et al., 2017). As they experienced severe human rights violations, including human trafficking and intense abuses (financially, psychologically, physically, and sexually), their desperate attempts to overcome adversities (individual and familial) and vulnerability, ultimately compounded their overall health risks (Chongsuvivatwong et al., 2011). For instance, during the pandemic, they were asked to work (or continue working) in areas of high risks/exposures (e.g., as cleaners, domestic workers) without sufficient protections, and they were placed in unhygienic and cramped accommodations with inadequate medical facilities. Such multipronged adversities not only increased their chances of infection (some described such living areas as epicenters of COVID-19) but also triggered mental-health issues among the marginalized workers (Gasana & Shehab, 2020).

During the pandemic, countless Bangladeshi migrant workers lost their jobs; such sudden joblessness and income uncertainties eventually affected migrant workers particularly when their families experienced hunger and impoverishment. Many of them, at home or in abroad, expressed hopelessness for having no promise of any employment and no means of supporting their loved ones, in the near future. Thus, the income insecurities essentially affected their overall health and well-being, which was evident from their articulations; for instance, according to some migrants, losing their jobs was way more horrifying for them than COVID-19 death.

The migrant workers, in Facebook, mentioned that their financial and psychological stress further worsened because of several contextual factors, including fear of government restrictions and deportation, physical and mental abuses (especially those who worked in domestic sector), economic burden for doing testing (often mandatory) and treatments, and for unanticipated price rise of essential commodities by opportunist businessmen. Consequently, being capacitated to withstand such stress, young Bangladeshi workers exhibited desperation; a few of them tried to/committed suicide. Another factor contributed hugely, both socially and religiously, was their inability to participate/attend the last rites of the deceased. Importantly, the bodies of many migrants remained in the hospitals indefinitely, which prevented the friends and families from performing funerals.

Scholars have noted that economically impoverished migrant workers to the Middle East from South Asian countries were “at a high risk of mental illness due to their living and working conditions” (Adhikary et al., 2011). They further observed that depression, anxiety and posttraumatic stress were some of the key components of their mental health conditions (Adhikary et al., 2011). On the top of that, the outbreak of pandemic posed additional stress to the migrant workers, which further deteriorated their overall psychological well-being (Alahmad et al., 2020). As the pandemic situation exhibited possibilities of economic downturns both in the short- and long-run, the financially uncertain migrant workers became more vulnerable as they failed to envision any sign of revival in the near future, which ultimately yielded high mental stress among them.

Amid such stressful scenarios, migrant workers earnestly and collaboratively tried to overcome their adversities both individually and collectively. For example, some migrants, with their finite financial capacities, organized social events (e.g., celebration of religious festivals such as Eid, and birthdays) and tried their best to support underserved workers—materially, psychologically, and communicatively. In other words, human connection and cooperation emerged as foundational to such community-led initiatives for survival; where human communication (and agencies), one of the pivotal aspects, emerged both as the means and ends for social organizing toward ensuring survival at the margins.

Future research might benefit from incorporating lived realities of marginalized communities from other parts of the global South by focusing on a variety of identity markers including nationality, race, ethnicity, and gender. Once this pandemic is over, more data will be available, which would help us to draw a more grounded picture of the migrants’ realities. Data could be gathered beyond the March’20—August’20 window, and we would be able to conduct face-to-face in-depth interviews, which would potentially enrich future research initiatives.

In understanding the lived realities, negotiations, and endeavors of the migrant workers, the critical lens aided us to investigate the issues of access, resource scarcity (both material and communicative), human rights, policies, disparities, and inequalities. Thus, such critical communicative engagements helped raise the voices and agencies of the underserved communities to create avenues for bringing about social justice and equity at the margins.

## Notes

1 The molecular structure of this virus, under a microscope, looks like a crown, and hence the virus became known as “coronavirus.” The Latin word corona means a crown. The virus is more commonly known as COVID-19 globally, where the “CO” stands for “corona,” “VI” stands for ‘virus,’ and the “D” stands for “disease.”
